# Food and social complexity at Çayönü Tepesi, southeastern Anatolia: Stable isotope evidence of differentiation in diet according to burial practice and sex in the early Neolithic

**DOI:** 10.1016/j.jaa.2013.01.002

**Published:** 2013-06

**Authors:** Jessica Pearson, Matt Grove, Metin Özbek, Hitomi Hongo

**Affiliations:** a Archaeology, Classics and Egyptology, Hartley Building, University of Liverpool, Liverpool L69 3GS, UK; b Department of Anthropology, Hacettepe University, Ankara, Turkey; c School of Advanced Science, Graduate University for Advanced Studies, Shohan Village, Hayama, Kanagawa 240-0193, Japan

**Keywords:** Neolithic, Near East, Social differentiation, Burial practices, Isotopes, Diet

## Abstract

The identification of early social complexity and differentiation in early village societies has been approached in the past most notably through the evaluation of rituals and architectural layouts. Such studies could be complemented by an approach that provides data about everyday behaviours of individuals. We took 540 human and animal bone samples for stable carbon and nitrogen isotope analysis from the Neolithic site of Çayönü Tepesi in southeastern Anatolia. The inhabitants at this site chose to bury their dead in two different ways at different times during its occupation: beneath the floors of their houses, but also inside a public mortuary building known as the Skull Building. This variation provides an opportunity using isotope methods to test whether there was evidence for structuring of daily activities (diet in this case) that might serve to reinforce this change in burial practice. We show that when the inhabitants of Çayönü Tepesi changed their architecture and operated different burial practices in conjunction, this coincided with other aspects of behaviour including socially-constituted food consumption practices, which served to reinforce social identities.

## Highlights

► People from secondary burials (Skull Building) had different diets to those buried beneath houses. ► In the later Cell Plan sub-phase of occupation males and females had different diets. ► Dietary variation resulted from different types and/or amounts of meat and plant protein. ► The isotope results follow sex and burial practice suggesting food reinforced social behaviours.

## Introduction

One of the central issues in the understanding of early social organisation and the origins of social complexity in early communities is whether different burial practices were the result of incipient hierarchical social structures that singled out individuals for specific roles, or acknowledged differences between groups that were organised along more egalitarian lines (Binford, 1971; Carr, 1995; Goldstein, 1981; Hodder, 1982; Kroeber, 1927; Kuijt and Goring-Morris, 2002; Peterson, 2010; Ucko, 1969). It has been argued, on a number of occasions, that burial practices of the Neolithic Near East, and in particular those of the Pre Pottery Neolithic B (PPNB) (9300–8500 bp) communities in the Levant and elsewhere, were inclusive events able to combat social tension caused by population increase and uncertainty in resource security, thereby allowing for the successful negotiation of rapidly changing economic, technological, social and political regimes (Kuijt, 2000). Others have argued that some form of hierarchy existed in this period and penetrated many aspects of life, such as the production of figurative art and burial of the dead within the house (Rollefson, 2000 see also Garfinkel, 1988 on the use of plaster). Goring-Morris (2000) argues that since the less common practices, such as skull modification and decoration, cross sex and age boundaries, they are more likely to be representative of socio-political differentiation based on inherited status, which served as a mechanism for orderly transfer of accumulated power and wealth upon death of an individual. However, he also points out that the extent to which these secondary burial practices reflect the status of the individual pre-mortem is debatable (see also Byrd and Monahan, 1995 on the Natufian) and difficult to confirm independently.

## Aims and objectives

Although burial practices can bear some relationship to an individual’s role in life, these practices are rarely passive reflections (Hodder, 1982). Such approaches wrongfully assume that all practices (or the inclusion of particular objects) mean the same thing to all individuals or groups and that changes in social developments are translated in burial practices, which have been shown not to be the case amongst ethnographic studies of the Nuba of the Sudan (Hodder, 1982) or the Merina of Madagascar (Bloch, 1971) for example. Any attempt to identify what in-life roles people that were buried in a particular manner had requires an approach that provides data about everyday behaviours of the individual rather than associated artefacts or the archaeological features of burial. These have been somewhat limited to date, although physical anthropology has offered some solutions: Fletcher et al. (2008) showed, through radiographic assessment of a plastered skull from Jericho, that evidence of skull modification was present but not visible by eye, thus suggesting that the process in childhood was the important factor, rather than the outcome of a visibly manipulated skull. Observation of limb bones at Çatalhöyük, Ain Mallaha and Abu Hureyra (Eshed et al., 2004; Molleson, 1994; Molleson et al., 2005) revealed evidence of occupational pathologies caused by physical activity necessary to undertake tasks such as grinding cereals for flour. Although this last approach tells us which individuals were involved in resource collection and preparation, crucially, it does not tell us who actually consumed resources.

It is with these issues in mind that an extensive programme of stable carbon and nitrogen isotope analysis was undertaken from Çayönü Tepesi, a Neolithic site in southeastern Anatolia. This site was chosen since both primary burial beneath house floors and mass secondary burial in a public building was used. Isotope signatures in bone provide long-lived information about food consumed. Therefore, if human diet consisted of carefully structured meals that were eaten over a number of years, we can compare this information among individuals afforded a particular type of burial to see if they were repeatedly eating or not eating certain foods in life. Any differences may have been due to participation or exclusion in certain meals/events or if food was distributed in a highly formalised fashion to groups with strict rules governing sharing. For the isotope analysis, we sampled 286 humans and 254 animals to reconstruct diet of the individual adult inhabitants. Specifically, we address two questions: firstly, whether individuals that were afforded secondary burial treatment in the public building in the middle sub-phases of occupation had similar diets or not to those buried (and left) as primary inhumations beneath houses in the preceding and following sub-phases. Mass burial in a public building would have been a more public affair, so if diets that differed according to burial practice did so independently of changes in resource availability over time, this would suggest that burial practices changes were accompanied by diet changes in order to reinforce social identity and further promote cohesion. Conversely, differential access to resources amongst people buried in the same way could have fuelled social conflict that already existed from uncertainty about food security and pressure from population expansion. A second question is whether the diet was similar for all individuals during the sub-phases when primary burial was used, or if the diet varied according to sex (thereby confirming observations of dental pathologies), rather than an inherited or achieved status. Such a distinction would provide an early indication of social differentiation in terms of food consumption and support evidence from occupational pathology seen at other sites that early Neolithic communities regularly differentiated between groups in life.

## Background to the Pre Pottery Neolithic and burial practices

The Pre Pottery Neolithic was a period of extensive transformation; environmental conditions fluctuated, a number of plants and animals were domesticated, communities expanded in size and density, and the number of settlements increased (Kuijt, 2000; Özdoğan, 1997). As part of this change, mobile hunter–gatherer groups built more permanent structures where they tended to live year round transforming themselves into more sedentary villagers. It seems likely that such transformations caused changes in access to, and encroachment of, territories and put pressure on a range of resources, which contributed to social tension (Akkermans and Schwartz, 2003).

At the same time the explosion of ritual and florescence of symbolism is witnessed, which has been argued repeatedly as a counter-measure to reduce social tension caused by the new ways of living. The peak expression of symbolic and ritual behaviours occurs during the PPN and it has been argued that the range of secondary burial practices become common as early Neolithic societies used body part removal to mask social inequality through the emphasis of shared worldviews, that is effectively smoothing over social relations that caused tension amongst inhabitants (Hodder, 1982; Kuijt, 1996, 2008; Verhoeven, 2002). Burial practices of the PPN typically involved the removal of body parts, multiple burial of disarticulated individuals and differential (and varied) treatment of the skull in particular, which some scholars have suggested is evidence of skull cults linked to ancestor worship (Arensburg and Hershkovitz, 1989; Bienert, 1991; de Contenson, 1971) and more recently headhunting (Testart, 2008). These practices are found frequently across the Levant at sites such as Jericho (Kurth and Rohrer-Ertl, 1981), ‘Ain Ghazal (Rollefson, 1986), Kfar Hahoresh (Goring-Morris, 2000) and Nahal Hemar (Bar-Yosef and Alon, 1988) to name a few. Some aspects of these practices, with the exception of cranial modification, are also found beyond the Levantine corridor (and beyond the PPNB) in Turkey at sites such Çatalhöyük (Hodder, 2004), Boncuklu Höyük (Baird, forthcoming; Pearson, forthcoming), Çayönü Tepesi (Özdoğan, 1999), Domuztepe (Carter et al., 2003), Köşk Höyük (Özbek, 2009; Silistreli, 1988), and in Syria at Abu Hureyra (Moore and Molleson, 2000) Bouqras (Meiklejohn et al., 1992) and Jerf al Ahmar (Stordeur et al., 2000). Practices at these sites most frequently involve cranial removal and the burial of isolated skulls. Plastered skulls are only rarely encountered.

Discussion of the question of social complexity or differentiation and recognition of group identities amongst the presumed egalitarian communities of the Neolithic has focused mainly on ritual behaviours (such as secondary burial practices and other ritual deposits) (Goring-Morris, 2000; Kuijt, 2000) and also households through consideration of architecture (Byrd, 2000). Using burial practices as a method of identifying social differentiation is problematic in the absence of information about the living status of the individual because, as mentioned earlier, ritual and symbolic behaviours rarely represent faithful replications of everyday life (Hodder, 1982; and discussion by Fletcher et al. (2008)) and are used to re-establish social harmony by inverting social distinctions in life (or by providing opportunities for this to occur).

Physical anthropology can be a useful complement to approaches developed from material culture and such studies have been used to good effect in the Neolithic Near East, even if they have not always been considered alongside existing debates of ritual, burial practice or household organisation. Pilloud and Larsen (2011) studied the relationships between individuals buried at several central Anatolian sites to address biological versus social-based kinship, Molleson (1994, 2005) and Eshed et al. (2004) have shown evidence of division of labour by modifications of individual skeletons through various tasks and practices pointing to differential allocation of food production and preparation tasks (Eshed et al., 2004; Molleson, 1994; Molleson et al., 2005) as predicted by Rosenberg and Redding (2000) and Byrd (2000). So far, the only approach that has provided evidence of how food resources were shared, once the production and preparation had taken place, is palaeodietary reconstruction through stable carbon and nitrogen isotope analysis (Lösch et al., 2006; Pearson, in press; Richards et al., 2003). For this reason we focus here on stable isotope analysis because, like occupational pathology, it provides a window into individual and group identities but crucially goes one step further by providing direct evidence beyond preparation and production of food, to how food is shared over the long term. As stable isotope measurements are carried out on individual skeletons, some can be singled out from others and grouped together according to biological or archaeological characteristics (i.e. sex or burial practice) (e.g. Barrett and Richards, 2004; Bentley et al., 2007).

## Çayönü Tepesi

**Fig. 1:**
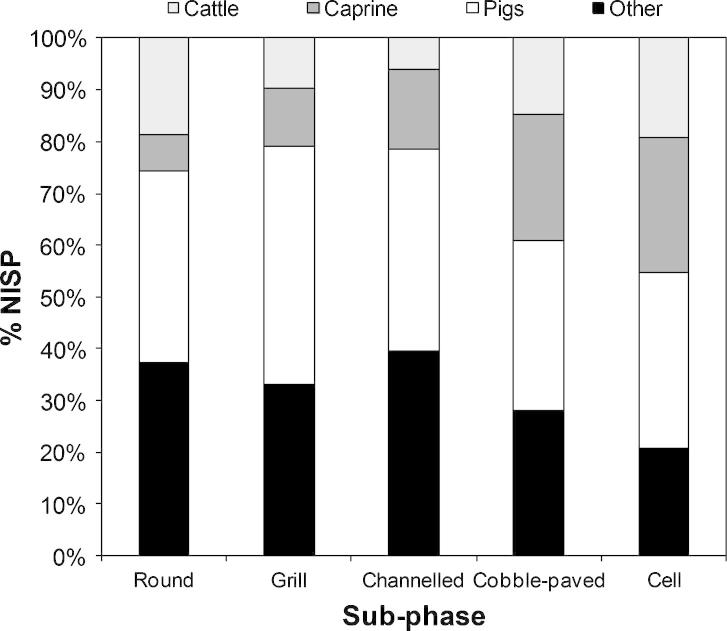
Relative proportion of taxa by sub-phase at Çayönü Tepesi according to %NISP (after Hongo et al., 2009).

Çayönü Tepesi is located on the Ergani plain in southeastern Turkey, approximately 40 km northwest of the modern city of Diyarbakır (Fig. 1). Halet Çambel and Robert Braidwood discovered the site during the 1963 Southeast Anatolia Survey and later excavated it with the aim of understanding the transition from hunting and gathering to farming (Çambel and Braidwood, 1980). Sixteen seasons of excavation were conducted between 1964 and 1991 (the latest years were directed by Mehmet Özdoğan and Aslı Erim Özdoğan), which uncovered over 4500 m^2^ of archaeological deposits spanning the early Neolithic to the Middle Ages (Özdoğan, 1999). The site is perhaps best known for its Aceramic Neolithic deposits which date from the 9th millennium to the end of the 7th millennium cal BC (Pre Pottery Neolithic A (PPNA) to the final Pre Pottery Neolithic B/PPNC in Levantine terminology).

The sub-phases of Neolithic occupation at Çayönü Tepesi are characterised by shifts in building layout over relatively short periods of time. The six Aceramic Neolithic sub-phases are tabulated below (Table 1). Much of the architecture within the sub-phases is domestic, although a number of public buildings and spaces are also associated with specific sub-phases (Özdoğan, 1999). The most notable of these is the Skull Building, so-called because of the large numbers of disarticulated skulls and limb bones of both sexes and juveniles found buried there (Özbek, 1988a). This structure seems to have been long lived, witnessing numerous episodes of rebuilding, and where as many as 400 individuals were interred throughout its use. During the first phase of use (BM1), which is contemporary with the Round and Grill building sub-phases, approximately 120 secondary burials have been recovered (Özdoğan and Özdoğan, 1998). At the same time approximately 65 burials were deposited beneath contemporary domestic houses of the Round and Grill plan sub-phases. Most of the human deposits (*n* = 280) found in the Skull Building were interred during the later BM2, which is phase contemporary with the middle sub-phases of occupation (Channelled and Cobble-Paved sub-phases) (Özdoğan and Özdoğan, 1998) and correlates approximately with the early–middle PPNB (Özdoğan, 1999). Primary burials were very rarely encountered in the Skull Building, numbering only one or two (Özdoğan, 1999). Secondary burial beneath contemporary houses was significantly less (c. 30) and are represented only by small fragments of teeth or skull. The substantial nature of the building and the extensive deposits of human remains suggest it was an important structure for the inhabitants at Çayönü Tepesi. This public building was used to bury the dead as secondary inhumations until the onset of the Cell Plan sub-phase, when the inhabitants returned to burying their dead as primary inhumations beneath the floors of their houses (*n* = 135). Secondary inhumations were rarely encountered beneath houses.

The scale of secondary burial and the decision to bury individuals away from the house at Çayönü Tepesi is unique in the Near East. The reasons why these activities were performed on such a scale in a public building has been little discussed and a full consideration of this is difficult to discuss here. We use the data presented here to address the arguments of scholars such as Rollefson (2000) and Goring-Morris (2000) that secondary burial practices were afforded to individuals with particular socio-political status but whose in life status is otherwise difficult to establish. This then allows us to contribute towards the debates of scholars such as Kuijt (1996, 2000), Verhoeven (2002) that secondary burial activities were in some ways linked to attempts to mask social inequality attributed to greater pressure on communities caused by rapidly changing economic, technological social and political regimes.

### Faunal and botanical evidence at Çayönü Tepesi

During the Neolithic the local vegetation surrounding Çayönü Tepesi comprised woodland/forest steppe with little access to aquatic resources. The inhabitants, many of whom probably occupied the site throughout the year, exploited wild (and possibly cultivated) pulses and nuts (almond, terebinth, pistachio), peas, lentil and bitter vetch rather than cereals, which, where present, are represented by wheat (*Triticum* spp.) predominantly (van Zeist and de Roller, 1992). The ratio of pulses to cereals varies throughout the occupation. Until the end of the Channelled sub-phase the cereal:pulse ratio was 1:3, cereals decrease in importance with the onset of the cobble-paved sub-phase (1:10) and increase again in the Cell Plan sub-phase (1:6).

The faunal remains are dominated by pigs (and wild boar) followed by caprines (mostly sheep), cattle and red deer. The faunal remains have been studied extensively and generally indicate that the Çayönü people exploited some domestic animals, exploited animals in the process of domestication and continued to hunt the same species in the wild (Ervynck et al., 2001; Hongo et al., 2002; Hongo et al., 2009; Hongo and Meadow, 2000; İlgezdi, 2000; Öksüz, 2000). There is some variation in the species exploited as shown by the Number of Identified Specimens (NISP) percentages (Fig. 1). Fig. 1 shows that the importance of *Sus* initially increases, after the Round building sub-phases, but then declines over time , caprines gradually increase in importance, cattle decrease in importance between the Round and Channelled sub-phase and then increase again in the Cobble-Paved and Cell Plan sub-phases. The decrease in cattle exploitation seems to have been mitigated by a corresponding increase in red deer exploitation (Hongo et al., 2009), which are included in the ‘Other’ category. Other wild taxa in this category include gazelle, which remain a constant component of the assemblage in the early sub-phases and gradually decline in number after the Channelled building sub-phase.

### Human diet and health at Çayönü Tepesi

Detailed reports of the health of the inhabitants buried beneath houses in single inhumations are presently confined to the dentition. The Skull Building individuals are currently being studied and these data are not yet available (A. Özdoğan, pers. comm.). Preliminary observations by Özbek (1988b, 1995) indicate that the inhabitants display the following pathological conditions: cribra orbitalia, porotic hyperostosis (both blood disorders often associated with iron deficiency), osteoarthritis and osteomyelitis (an infective disorder of bone). Examination of the oral health revealed that less than 5% of the examined permanent teeth revealed a carious lesion. Linear enamel hypoplasias were found at a rate of 4% in permanent teeth and 3% in deciduous teeth. However, ante-mortem tooth loss was found at a rate of 28% and dental wear patterns indicate heavy attrition, consistent with grit introduced into the diet from food processing technology, or foods that were already coarse and abrasive, even amongst young adults (15–20 years) and therefore the overall rate of lesions may have been higher in life. Dental abscesses were found in 63% of cases and correlated with heavy dental wear that exposed the pulp cavity on remaining teeth. Instances of periodontitis (gum disease) seemed to correlate with sex since 67% of males presented this pathology compared with 33% of females (Özbek, 1995). This suggests a sex division concerning consumption of cereals by males rather than females, who were assumed to have consumed more of the less cariogenic legumes that dominate the archaeobotanical record at the site. Infant mortality was high at birth, as was childhood mortality in the 3–4 year age group. Extended exclusive breastfeeding practices may have played a role in childhood deaths (Pearson et al., 2010).

## Isotope analysis and diet reconstruction

Isotope analysis of body minerals (apatite) provides dietary information that reflects whole diet (proteins, fats, and carbohydrates), whereas measurements of bone protein (collagen) reflect just the protein consumed. Each isotope also provides different information about the diet: nitrogen and carbon isotope ratios can be measured in collagen to ascertain the source of dietary protein (plant versus animal) and hence position in the food chain. Studies of bone apatite for whole diet measure carbon isotopes often alongside oxygen isotope ratios since body minerals contain very little nitrogen. Each method therefore provides a complementary view of diet. For this study we focus here on carbon and nitrogen isotope analysis of bone collagen, but acknowledge that a useful follow up study would be the analysis of bone apatite to measure whole diet.

The phrase ‘You are what you eat’ reflects the basic principles behind stable isotope ratios as palaeodietary indicators because they are assimilated in the body through the food chain (DeNiro and Epstein, 1978, 1981). Since bone collagen is an archive of dietary protein over at least 10 years in an adult, isotope analysis is a particularly useful source of information about dietary variations because any difference detected amongst stable isotope ratios laid down in bone collagen is the result of food repeatedly eaten on a regular, perhaps daily basis, rather than one-off meals on special occasions such as feasts (Pearson, in press). Given the highly structured nature of food consumption that would have to occur to produce distinctive patterning between groups at the same site, few sites have been able yet to demonstrate differences between sexes or burial practices in the Neolithic Near East (e.g. Lösch et al., 2006; Richards et al., 2003).

### Sources of carbon and nitrogen stable isotopes in the food chain

Nitrogen enters at the base of the food chain through plants, as either adsorbed nitrogenous compounds in soil (practised by most plants) or fixed directly from atmospheric nitrogen through symbiotic bacteria (a method used most notably by legumes) and is incorporated into bone with a fixed enrichment factor of ∼4‰ (per mil.) known as the ’trophic level effect’ (Ambrose and Norr, 1993; DeNiro and Epstein, 1981; Hedges and Reynard, 2007). Since this enrichment factor is more or less constant in the bone collagen between the different positions in a food web, the nitrogen isotope value of individuals can be used to understand the relationship between consumers in the food chain (DeNiro and Epstein, 1981). As a very general rule, within a single population, the higher the nitrogen isotope value (δ^15^N) the higher up in the food chain a member is likely to be and the more meat they probably ate.

Carbon also enters the food chain through plants during photosynthesis and is assimilated into the body of the consumer. Variations in plant stable carbon isotope ratios are mainly influenced by their physiology (O’Leary, 1981). C_3_ plants typically measure between −34‰ and −22‰ whereas C_4_ plants range from −20‰ to −7‰. Although some overlap does occur between CAM and C_4_, it is still possible to distinguish between plants following C_3_ versus C_4_/CAM pathways. Carbon is also used further up the food chain to distinguish between terrestrial and marine resources. Freshwater aquatic resources are more difficult to distinguish in the food chain than marine aquatic ones. Freshwater fish and birds are rarely measured in other Neolithic Anatolia sites. At Boncuklu Höyük (Central Anatolia) these resources ranged from −22.6‰ to −19.7‰ (2.9‰ range) and 7.8‰ to 12.7‰ (4.8‰ range) in nitrogen for fish and −23.1‰ to −17.2‰ (5.9‰ range) in carbon and 5.2‰ to 11.1‰ (5.9‰ range) in nitrogen for aquatic birds (Pearson, forthcoming). The rarity of freshwater resources on the site indicates that such foods are unlikely to have made a sustained contribution to human diet (Hongo et al., 2009). The carbon enrichment factor between plants and a consumer bone is 5‰ and just 1‰ between herbivore and carnivore bone (DeNiro and Epstein, 1978). A review of the archaeobotanical data at Çayönü Tepesi has shown no evidence for the physical presence of C_4_ or CAM plants (see Table 1, Hongo et al., 2009), although they cannot be completely discounted on this basis because animals may have fed on their leaves, which would leave no physical trace in the archaeobotanical record.

### Environmental variation in carbon and nitrogen isotope ratios

The δ^13^C ratios of terrestrial higher plants have been shown to vary considerably (e.g. Glagoleva and Chulanovskaya, 1992; Heaton, 1999; Tieszen, 1991; Tieszen and Fagre, 1993). Plants respond to the relative availability of water (water-use efficiency) during photosynthesis by limiting water lost as vapour during photosynthesis in warm conditions. How they respond affects the carbon isotope ratio of their tissues. Plants growing in areas of abundant water have low water-use efficiency, and consequently more negative carbon isotope ratios. Nutrient impoverishment (lack of nitrogen or phosphorus) and exposure to low temperatures for C_3_ photosynthesis also result in more negative carbon isotope ratios (Tieszen, 1991). C_3_ plants occupying areas with low water availability increase their water use efficiency, which causes their carbon isotope ratios to be more positive (Araus et al., 1997, 2001; Stewart et al., 1995). Water deficits can be caused by low rainfall or higher temperature and can be mimicked by the presence of saline rather than fresh water. The range in carbon isotope ratios is much larger for aquatic systems (both freshwater and marine environments) than terrestrial ones. Çayönü Tepesi is located approximately 300 km from the nearest coast (Black Sea), which allows us to exclude any dietary contribution from marine resources. The site is, however, situated in the Tigris river valley close to a tributary although there is no evidence of marshland in the immediate vicinity and little evidence for freshwater fish in the faunal remains (Hongo et al., 2009) suggesting that the impact of this aquatic system is likely to be small.

Nitrogen also enters the food chain either as adsorbed nitrogenous compounds in soil or fixed directly from nitrogen in the atmosphere. Nitrogen isotope signatures in a food web are additionally influenced by a large number of environmental factors most of which can cause ratios to rise. In plants these include aridity (e.g. Ambrose, 1991; Cormie and Schwarcz, 1994; Gröcke et al., 1997; Heaton, 1987; Schwarcz et al., 1999) and soil salinity (e.g. van Groenigen and van Kessel, 2002; cf. Handley and Scrimgeour, 1997; Robinson et al., 2000). More recently, Bogaard et al. (2007) and Fraser et al. (2011) have suggested that manuring of crops introduces heavy nitrogen into the food chain resulting in higher ratios of manured-crop consumers. Çayönü Tepesi shows no evidence for an intensive agricultural regime, so this potential source of input is not relevant. Animal physiology also plays a role in pushing up nitrogen isotope ratios in tissues. Minimising the amount of water lost in urine (which contains nitrogen-rich urea) in warmer climates (for a discussion of this see Ambrose, 2000). Ruminating animals (Caprines, cattle, deer and others) normally have higher nitrogen isotope ratios than non-ruminants (most notably equids and pigs) (Cormie and Schwarcz, 1996; Macko et al., 1982; Sealy et al., 1987; Wattiaux and Reed, 1995). Similarly, nitrogen isotope ratios can be raised during starvation and protein deficient diets, which causes urea nitrogen salvaging by the body (e.g. Ambrose, 2000; Barboza et al., 1997; Fuller et al., 2005).

While there is ample information about the nitrogen isotope values of many archaeological animals across the globe, the difficulty measuring proteins (rather than bulk tissue) of plants has meant that our knowledge of archaeological plant nitrogen isotope ratios is still very limited. Ecological studies of modern terrestrial plants report than most plants generally range in nitrogen between −6‰ and +5‰. However, effects of volatilisation, nitrification and denitrification in soils (Handley et al., 1999) have been known to produce plant tissues less than −10‰ and greater than 10‰ (Finlay and Kendall, 2007). Higher ratios would normally be associated with wetland environments or anthropogenic disturbance.

## Methods

The human remains from Çayönü Tepesi are housed in the Department of Anthropology, Hacettepe University, Ankara. Destructive sampling of the Çayönü Tepesi human remains collection was only permitted on ribs. No other elements such as skulls or long bones could be sampled. Age and sex determinations and details of the demographic profile have been reported previously (Özbek, 1988a,b, 1995). The sex of adults was assigned, where possible, following Ferembach et al. (1980) while age stage was assigned following Ubelaker (1989). A total of 540 bone samples were taken from Çayönü Tepesi consisting of 286 from human bone and 254 from animal bone covering the period of Aceramic Neolithic occupation. Samples were taken from caprines, cattle, cervids, equids, pigs and gazelle across the Aceramic phases from Round to Large Room (6 sub-phases). Measuring samples across all sub-phases allows human diet to be reconstructed for each sub-phase, but also allows monitoring of fluctuations that may be associated with palaeoenvironmental change. We aimed to take 10 samples of each species from each phase (a potential total number of specimens of 360), however, samples were not always available in such numbers from the rarer species. All human remains were sampled including the secondary/multiple burials in the Skull Building, for which a specific methodology was adopted to exclude potential duplicate sampling (see below).

Collagen was extracted from approximately 300–500 mg of compact bone using a modified Longin method (1971) described elsewhere (Pearson et al., 2007). Measurements were made at the Research Laboratory for Archaeology and the History of Art, Oxford University, UK on a Europa Geo Gas Chromatography Continuous Flow–Isotope ratio monitoring mass spectrometer (GC–CF–IRMS). All samples were measured in duplicate machine runs and are reported drift-corrected. All reported samples fell within the range for well-preserved collagen established by Ambrose (1990). Both isotopes are reported relative to the AIR for nitrogen and vPDB for carbon. The error on measurements of both isotopes is ±0.1‰ (Pearson, 2004). A total of 121 (∼22%) samples failed to yield sufficiently well preserved collagen, including all but four from the initial phases of the Skull Building use (BM1). Since the BM1 data is very small, these data are not discussed separately here. Samples from the human juveniles relating to weaning practices are presented elsewhere (Pearson et al., 2010).

Since the deposits of human remains in the Skull Building are disarticulated it is possible that samples taken may be multiple samples from the same individuals. Therefore, two sub-sampling methods were used (once the isotope measurements were known) to detect samples most likely to be duplicates. These subsampling approaches allow for the assessment of any confounds or biasing effects created by inclusion of duplicates in the dataset, as well as providing more robust comparisons between the skeletal samples from the domestic and Skull Building datasets. The first subsampling method involved using only the first sample from any given archaeological feature and discarding the others; at *n *= 47, this ‘FirstSamples’ subsample is considerably smaller than the ‘AllData’ sample at *n *= 108. Secondly, the ‘Euclidean’ subsample (*n *= 67) was created by first calculating pairwise Euclidean distances between the individual samples in the FirstSamples subsample when these were graphed as a bivariate plot of δ^13^C‰ against δ^15^N‰. The distribution of these distances was then calculated and the value at the 50th percentile noted. Samples from each archaeological feature in the AllData sample that were separated by less than this value (=0.90068) were then excluded from the analysis. This subsampling method employs the rationale that, to be plausibly counted as belonging to different individuals, skeletal elements sampled from the same feature should be at least as different as the median difference between skeletal elements sampled from different features (as the latter are known *a priori* to represent different individuals).

## Results of stable carbon and nitrogen isotope analysis

**Fig. 2:**
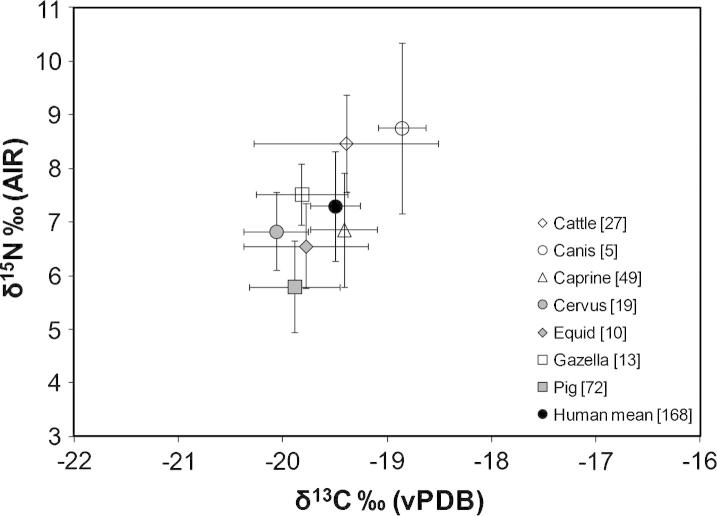
Carbon and nitrogen stable isotope data for all humans and fauna from all sub-phases at Çayönü Tepesi. Data are given as the mean and 1 sd (error bars). Potential duplicated humans in the Skull Building not removed.

Firstly, we consider the general human diet from all phases to determine the average diet at the site (Fig. 2). The adults from all sub-phases vary in δ^13^C from −20.1‰ to −18.4‰ (1.7‰ range) and in δ^15^N from 3.7‰ to 9.7‰ (6.0‰ range). These data show that all humans and animals had a C_3_-based terrestrial diet, which is based on the use of a specific Neolithic Anatolian C_3_ plant carbon isotope value of −23.0‰ (Pearson et al., 2007) rather than the lighter global average reported by others (e.g. O’Leary, 1981). The nitrogen isotope ratios are relatively low amongst both humans and animals compared to sites such as Çatalhöyük (Richards et al., 2003; Pearson, in press) or Aşıklı Höyük (Pearson et al., 2007, 2010) and are more similar to Nevalı Çori (Lösch et al., 2006). The inhabitants were not intensively rearing animals or agricultural cereals so sources of isotope variation relating to agricultural and herding practices (which normally result in higher than normal isotope ratios) described above are not relevant here.

**Fig. 3:**
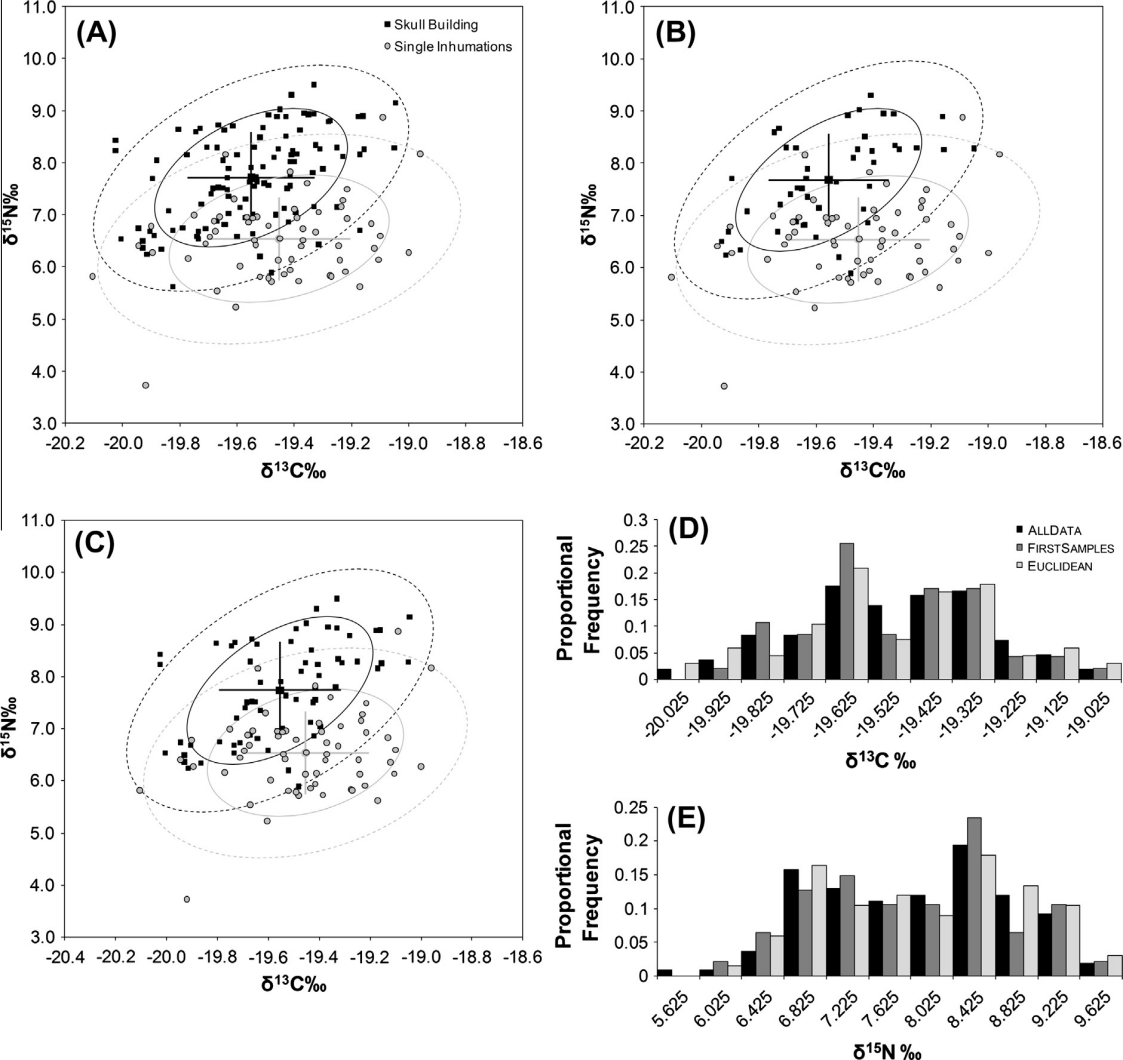
Carbon and nitrogen stable isotope data for all humans from the domestic buildings sample plotted for comparison with the Skull Building human subsamples: (A) AllData, (B), FirstSamples and, (C), Euclidean. The AllData subsample contains all of the samples measured including possible duplicates. The FirstSamples subsample contains only the first samples selected from a feature in the Skull Building. The Euclidean subsample was created by first calculating pairwise Euclidean distances between the individual samples in the First Samples subsample when these were graphed as a bivariate plot of carbon against nitrogen isotope ratios. The distribution of these distances was then calculated and the value at the 50th percentile noted. Crosses mark one standard deviation either side of the mean value for carbon and nitrogen; confidence ellipses for the means are plotted at 68% (solid lines) and 95% (dashed lines). (D and E) show the proportional frequencies of carbon and nitrogen measurements separately for the AllData, FirstSamples, and Euclidean subsamples, demonstrating that subsampling does not appreciably affect either the location or the shape of the distribution of the measurements of either isotope.

The variation in human δ^15^N is large (greater than one trophic level) suggesting a substantial difference between the amount of meat consumed by the inhabitants. The mean nitrogen isotope ratio of 6.8‰, and the similar (and often higher) nitrogen isotope ratios of caprines, cattle and gazelle suggests that protein from these animals made only a small contribution to the average diet and a higher contribution to the few individuals with isotope ratios much higher than this mean (the full range of human values is shown in Fig. 3). The mean nitrogen isotope ratios for pigs is 5.8‰ suggesting that this resource was the most likely animal protein consumed, although the 1‰ difference between the pig and human mean suggest that some people probably had very little animal protein in their diet from any species. Lösch et al. (2006) also noted low nitrogen isotope ratios at Nevalı Çori, which is not far (c. 100 km southwest) from Çayönü Tepesi. The range in human nitrogen isotope ratios is almost twice the variation observed at other Neolithic Anatolian sites including Nevalı Çori (Lösch et al., 2006) and recent work at Çatalhöyük (Pearson, in press). Since Çatalhöyük is a later and much larger site and the animals there were hunted and herded in a range of locations (Pearson, in press; Pearson et al., 2007), the Çayönü Tepesi data suggest that the range of foods consumed or the geographical range of where the resources were collected by the Çayönü Tepesi inhabitants may have been extensive.

Next, we compared the isotope ratios of individuals buried beneath domestic houses in the Round, Grill, and Cell building sub-phases with those buried in the Skull Building (BM2) during the main sub-phases of use (equivalent to the Cobble-paved and Channelled sub-phases). Results from Shapiro–Wilk tests demonstrated that not all sets and subsets of the Skull Building BM2 data were normally distributed; as such, nonparametric Mann–Whitney tests were used to test for significant differences between samples taken from within the Skull Building and those from single inhumations beneath the floors of domestic buildings. Tests were conducted using all the data from the Skull Building as well as using the two subsets as outlined above. The complete dataset from the domestic buildings was used in each test. Regardless of which Skull Building dataset was used, the values for both δ^15^N and δ^13^C were significantly different to those from domestic buildings indicating they consumed a different diet (Table 2 and Fig. 3).

### Nitrogen isotope ratios by species and phase

**Fig. 4:**
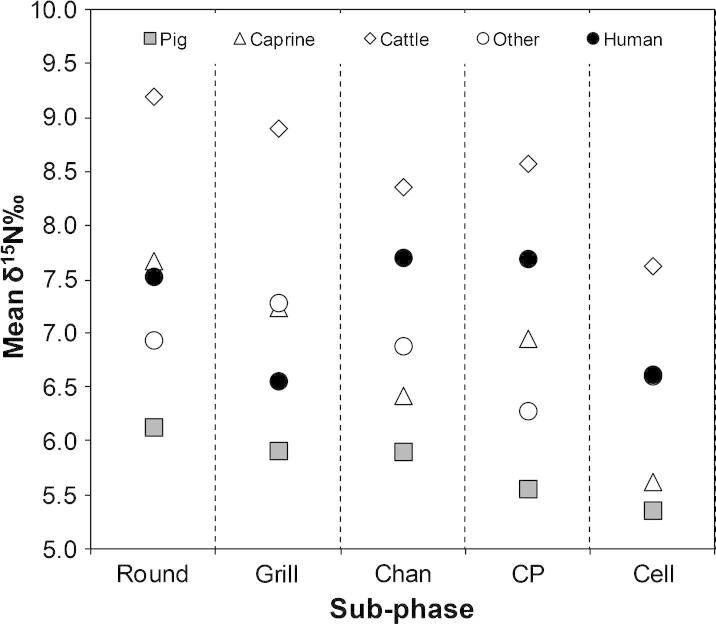
Mean nitrogen stable isotope data for individual taxa and humans by sub-phase at Çayönü Tepesi.

**Fig. 5:**
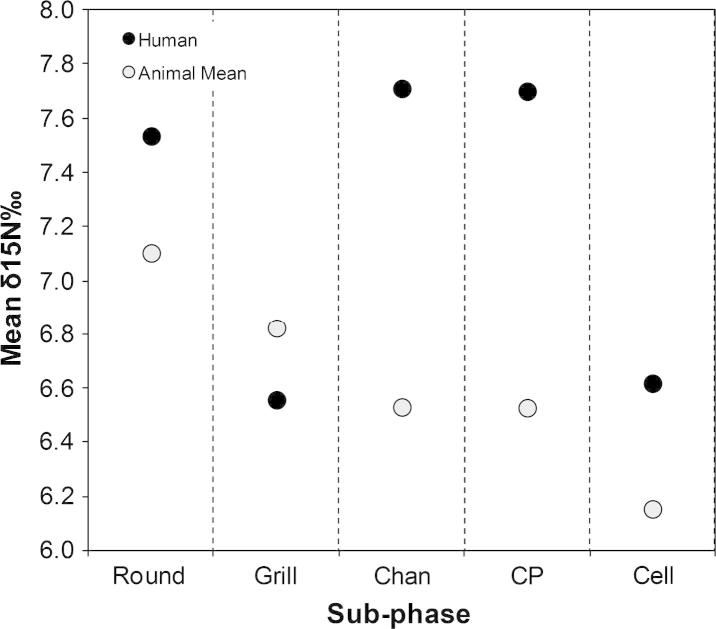
Mean animal (all taxa together) nitrogen isotope ratios plotted with mean human isotope ratios. The mean animal isotope ratio was calculated by incorporating their contribution to diet as determined by %NISP rather than averaging the four taxon groups given in Fig. 4.

Plotting the mean nitrogen isotope data by species and phase reveals some chronological trends (Fig. 4). Overall, the nitrogen isotope ratios show a gradual decrease from the Round to the Cell Plan sub-phase. Species of caprines and cattle show greater decreases than pigs and other taxa. When all animal species are averaged and compared with the nitrogen isotope ratios of humans over time (Fig. 5), the animals show a general decline in isotope ratios. This would appear to be related to environmental fluctuations rather than the result of human intervention since both domestic and known wild animals are affected. Since the human nitrogen isotope data shows an upturn in nitrogen isotope values and not the same downturn seen in the animals, this instead confirms the suggestion above that the individuals buried in the Skull Building during the Channelled and Cobble-paved sub-phase had a different diet than people buried under houses in the preceding Round and Grill plan sub-phases, and those in the later Cell Plan sub-phase.

### Human dietary reconstruction by mortuary area

**Fig. 6:**
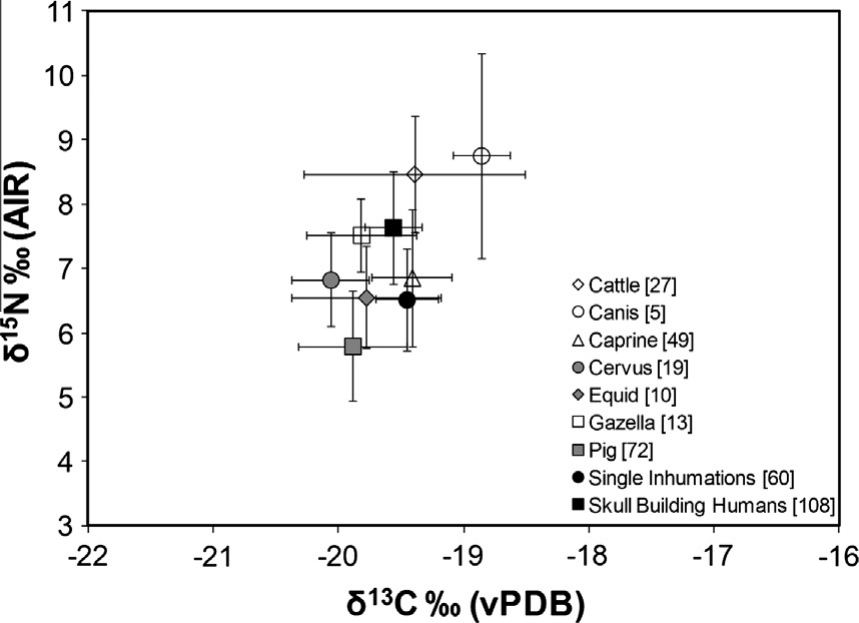
Carbon and nitrogen stable isotope data for all humans (separated according to burial at single inhumations beneath house floors and Secondary inhumation in the Skull Building) and fauna from all sub-phases at Çayönü Tepesi. Data are given as the mean and 1 sd (error bars). Potential duplicated humans in the Skull Building not removed.

The isotope ratios discussed above (Fig. 6) suggest that pigs (and wild boar) comprised the majority of the animal protein (when consumed) and caprines and cattle only made a small contribution to human diet, largely because a number of ruminants have higher nitrogen isotope ratios than humans. This suggests that foods with low nitrogen isotope ratios such as plants and some animals (perhaps small game such as hare) were important aspects of the diet of the inhabitants generally, and were the most important dietary sources for the individuals buried beneath houses in the Round, Grill and Cell Plan sub-phases who have the lowest nitrogen isotope ratios. The plants that are most likely implicated here would be legumes which normally have nitrogen isotope ratios closer to 0‰ than grasses and chenopods because they fix atmospheric nitrogen. In contrast, the higher nitrogen isotope ratios of the Skull Building individuals suggest the diet of people buried in this building included a contribution of leguminous plants supplemented by more caprines, cattle and gazelle protein and cereals, or simply more meat from boar/pigs as well as more modest portions from the ruminants and cereals.

### Male and female diet in the Cell Plan sub-phase

The Cell Plan sub-phase represents a relatively large population of skeletons where sex could be confidently identified (compared with the Round and Grill Building sub-phases where there are only a few individuals). The mean carbon isotope ratios for males from the Cell Plan sub-phase were −19.5‰ and 6.4‰ for nitrogen. For females, the carbon ratios are identical at −19.5‰ but the nitrogen isotope ratio is lower at 6.0‰. These data were analysed using Student’s *T*-Test (*p* = 0.046, *df* = 31), which shows the difference is statistically significant. These data suggest that males had access to either more pig/boar protein or to the resources with higher nitrogen isotope ratios (cattle, caprines and cereals) than females. These data suggest that the inhabitants at Çayönü Tepesi used food to differentiate between people within at least one sub-phase. Unfortunately, the lack of data on sex for the Skull Building individuals, and the low number of sexed adults from other sub-phases and house floors means that we cannot look to see if this mechanism of differentiation exists in other sub-phases or burial types at the site.

## Discussion

These data represent some of the earliest stable isotope evidence for the use of food to underpin social differentiation and complexity in the Near East. We show that the inhabitants of Çayönü Tepesi changed their architecture and operated different burial practices in conjunction with other aspects of behaviour including food consumption practices suggesting these were socially-constituted identities. Earlier evidence of the dental health at Çayönü Tepesi had already indicated sex distinctions in diet from the greater incidence of gum disease amongst males compared with females. This is supported by the isotope data for the Cell building sub-phase which suggests that males enjoyed more access to pig/boar protein and/or to more caprines, cattle and gazelle and protein from cereals compared to females. Unfortunately, the earlier levels could not be assessed so we cannot test if this was also true in other sub-phases of occupation.

It would seem that the maintenance of distinct social identities amongst the living was so important that they had to be frequently (and perhaps daily) reinforced through food consumption. Meat and cereals (which would have been relatively novel at the site) seemed to play a key role in these behaviours. Overall, we suggest that food consumption was part of the method used more regularly to alleviate social tension and mask social inequality alongside less frequent performances of feasting and burial of the dead. Since the Cell Plan sub-phase reveals evidence for sex differentiation in diet, a time when secondary burial practices were no longer the norm, our data would seem to suggest that differential access to food was not a cause of tension assuming that secondary burial was a method to alleviate this.

Returning to the hypothesis of Kuijt (2000) and others that secondary burial practices was a mechanism that promoted social cohesion, these data suggest that the more inclusive practice of burial of Çayönü Tepesi dead in the public Skull Building was accompanied by a specific diet throughout a significant period of adult life, which perhaps further served to reinforce social identity. Once the dead were buried exclusively beneath the houses in the Cell Plan sub-phase, a practice that would have been a much more private affair, sex-based food ways provide evidence of a different social behaviour that was more exclusive and points to early evidence of social complexity through social rules governing the distribution of resources among groups.

## Conclusion

The early Neolithic in the Near East has been frequently associated with some of the most compelling evidence for the beginnings of social complexity and ritual. However, the evidence typically used rarely seems to reflect everyday life. The role of more repetitive, everyday and normal activities and allocation of tasks and resources is much less studied. Through measurement of stable carbon and nitrogen isotope ratios, which allows the long term diet of people to be determined on an individual basis, we have shown how food resources were distributed at Çayönü Tepesi as a part of enduring and highly repetitive processes in life that mirrored symbolic social complexity of burial rituals in death.

## Figures and Tables

**Table 1:** Sub-phase chronology at Çayönü Tepesi with absolute and relative dating based on 39 radiocarbon dates (after Erim-Özdoğan, 2007).

Sub-phase	Absolute dating (bp)	Levantine period
Round Building	10,200–9400	PPNA
Grill Building (Early)	9400–9200	PPNA
Grill Building (Late)	9200–9100?	Early PPNB
Channelled Building	9100–9000	Early PPNB
Cobble-Paved Building	9000–8600?	Middle PPNB
Cell Plan Building	8600–8300	Late PPNB
Large Room Building	8200–8000?	PPNC

**Table 2:** Results of Mann–Whitney *U* tests for significant differences in human carbon and nitrogen isotope ratios between the domestic building and Skull Building samples at Çayönü Tepesi. The Skull Building data is divided into three groups: AllData subsample, which contains all of the samples measured including possible duplicates. FirstSamples subsample, which contains only the first samples selected from a feature in the Skull Building, and Euclidean subsample, which was created by first calculating pairwise Euclidean distances between the individual samples in the First Samples subsample when these were graphed as a bivariate plot of carbon against nitrogen isotope ratios. The distribution of these distances was then calculated and the value at the 50th percentile noted.

		*U*	*Z*	*p*
δ^13^C‰	AllData	2419.5	−2.566	0.010
FirstSamples	2142.0	−2.369	0.018
Euclidean	3773.0	−2.354	0.019

δ^15^N‰	AllData	1015.0	−7.269	<0.001
FirstSamples	460.5	−5.889	<0.001
Euclidean	2424.0	−6.466	<0.001
